# Association Between Fine Particulate Matter (PM_2.5_) and Severity of Acute Respiratory Infections Among Young US Children in the Major Cities in the United States: A Claims-based Cohort Study

**DOI:** 10.1093/ofid/ofaf442

**Published:** 2025-10-10

**Authors:** Damien Foo, Annette K Regan, Seulkee Heo, Eric B Schneider, Joseph Canner, Yimeng Song, Michelle L Bell

**Affiliations:** Yale School of the Environment, Yale University, New Haven, Connecticut, United States; Department of Research and Evaluation, Kaiser Permanente Southern California, Pasadena, California, United States; Fielding School of Public Health, University of California Los Angeles, Los Angeles, California, United States; School of Nursing and Health Professions, University of San Francisco, San Francisco, California, United States; Yale School of the Environment, Yale University, New Haven, Connecticut, United States; Yale School of Medicine, Yale University, New Haven, Connecticut, United States; Yale School of Medicine, Yale University, New Haven, Connecticut, United States; Yale School of the Environment, Yale University, New Haven, Connecticut, United States; Yale School of the Environment, Yale University, New Haven, Connecticut, United States

**Keywords:** acute respiratory infection, air pollution, antiviral medication, particulate matter, severity

## Abstract

**Background:**

Acute respiratory infections (ARIs) are a leading cause of morbidity and mortality among children. Air pollution may play a role in the exacerbation of ARIs via inflammation, immunosuppression, and oxidative stress, yet this effect has been infrequently evaluated among children.

**Objectives:**

Evaluate the impact of short-term exposure to fine particulate matter (PM_2.5_) to ARI severity among US children aged <5 years.

**Methods:**

We analyzed data from a claims-based cohort of children included in a private health insurance plan (Merative^™^ MarketScan^®^ Commercial Claims and Encounters database) who were diagnosed with an ARI between January 2018 and March 2020. We use daily monitored PM_2.5_ concentrations at the metropolitan statistical area level to estimate the short-term weekly PM_2.5_ exposure. We evaluated the association between short-term PM_2.5_ exposure and the risk of prescription claim for antiviral medication, hospital admissions and readmissions for an ARI, intensive care unit (ICU) admission for an ARI, mechanical ventilation, and length of stay among hospital-admitted and ICU-admitted children using generalized linear models.

**Results:**

The risk of an antiviral prescription claim increased by 11% (95% confidence interval, 1.07-1.15) per interquartile range increase in PM_2.5_ exposure (3.34 µg/m^3^); this association was consistent regardless of age, biological sex, and influenza vaccination status. We observed a 6% increased risk of ICU admission (95% confidence interval, 1.02-1.10) among children not vaccinated against influenza and no increase among vaccinated children.

**Conclusions:**

Short-term PM_2.5_ exposure may contribute to ARI severity among children. Influenza vaccination may modify the risk of severe ARI-associated outcomes.

## Teaser

Suggestive evidence that short-term exposure to PM_2.5_ increases the risk of severe acute respiratory infection outcomes in young children. Influenza vaccination status may modify the impact of short-term exposure to PM_2.5_ leading to milder respiratory illness in young children.

Acute respiratory infections (ARI) causes a serious health burden and are the second leading cause of death worldwide for children aged <5 years, contributing to 13.3% of deaths in 2019 [1]. Scientific evidence suggests that ambient air pollution exacerbates respiratory illnesses, including asthma, chronic obstructive pulmonary disease, and ARIs [2]. It has been hypothesized that long-term exposure to ambient air pollution, a major contributor to global disease burden [3], increases the risk of severe respiratory illness outcomes through multiple biological mechanisms.

Exposure to high levels of air pollutants, including fine particulate matter (PM_2.5_), in the respiratory tract causes inflammation, oxidative stress, and immunosuppression, which can increase susceptibility to infection and/or impair immune responses to infection, leading to more severe respiratory disease. Multiple studies have investigated the association between PM_2.5_ and respiratory infections. PM_2.5_ exposure is positively associated with influenza infection [4, 5], ARI [6–8], and influenza-like illness [9, 10] as well as severe respiratory illnesses, including hospital admission for respiratory infections in young children [11–13]. Young children may have inherently greater risk of severe respiratory infections compared to older individuals, which may be due to their narrower airways, developing lungs, and greater propensity for exposure to ambient air pollution [8]. Despite this, the role of air pollution on the severity of ARIs in young children is understudied [14–16].

We investigated the association between short-term PM_2.5_ exposure and key indicators of ARI severity, including prescription claim for antiviral medication, hospital admissions and readmissions, intensive care unit (ICU) admission, mechanical ventilation, and length of hospital stay among children aged <5 years using a large national claims-based cohort of children with a date of diagnosis for an ARI between January 2018 and March 2020.

## METHODS

### Study Design and Population

We conducted a claims-based cohort study including all children aged <5 years in the Merative^™^ MarketScan^®^ Commercial Claims and Encounters (hereafter referred to as the MarketScan) database. The MarketScan database is one of the largest longitudinal health insurance claims-based limited datasets in the United States. The database includes information on hospital inpatient, outpatient (including prescription claims), and emergency department encounters such as date of service, diagnostic, procedure, and pharmaceutical codes, length of stay, and demographic characteristics at the metropolitan statistical area (MSA) level. The study cohort consisted of children with an inpatient, outpatient, or emergency department record with principal diagnosis for an ARI (Supplementary Table 1) between 1 January 2018 and 31 March 2020 (before the COVID-19 pandemic) and who were continuously enrolled in a participating private health insurance plan from the start of the most recent influenza vaccine campaign in the contiguous United States (ie, September of the preceding year) through the end of the winter respiratory virus season (ie, March/April). Unique ARI episodes were considered those with ≥ 28 days between each episode. We estimated the date of onset for each unique ARI episode as 3 days prior to the first health care encounter for an ARI (Supplementary Table 1).

The Yale University Human Subjects Protection Program determined that this study was exempt from institutional review.

### Exposure Measurement

We obtained daily PM_2.5_ measurements from the US Environmental Protection Agency Air Quality System (https://www.epa.gov/aqs); these were collected by state, local, tribal, and federal air pollution control agencies from thousands of monitors nationwide. Daily average PM_2.5_ concentrations were calculated from all available monitors within each MSA in the contiguous United States. Using daily MSA-level measurements of PM_2.5_ concentration, we calculated the average PM_2.5_ concentration in the 7-day window prior to the estimated date of onset of infection based on the child's residential MSA. Lag structures were selected a priori based on previous research [5, 17, 18].

### Outcome Measurement

We assessed 5 binary (yes/no) outcomes as indicators of ARI severity: (1) presence of a prescription claim record for antiviral medication (ie, oseltamivir), (2) hospital admission, (3) hospital readmission (among hospitalized cases only), (4) ICU admission, and (5) requirement for mechanical ventilation. We additionally assessed 2 discrete continuous outcomes: (6) length of stay in hospital among hospital-admitted children (count) and (7) length of stay in hospital among ICU-admitted children (count). Binary outcomes were classified as “yes” if the outcome occurred within 28 days of the estimated onset of infection. Hospital readmissions were classified as “yes” if a subsequent hospital admission occurred within 7 days of the discharge from the previous hospital admission. We restricted hospital readmission to uncomplicated admissions with an initial hospitalization with a length of stay ≤ 7 days, which are more likely attributable to ARI. Diagnostic (International Classification of Diseases and Related Health Problems, Tenth Revision, Clinical Modification), procedure (Current Procedural Terminology, Healthcare Common Procedure Coding System), and pharmaceutical (National Drug Code) codes used to identify the outcomes are provided in Supplementary Table 1.

### Covariates

**Figure 1.**
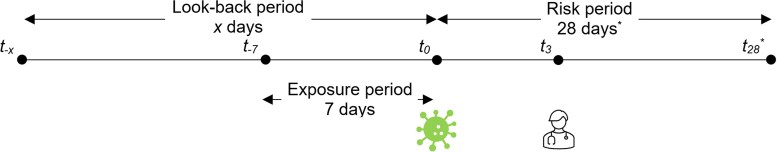
Timeline for assessing severe respiratory infection outcomes following fine particulate matter (PM_2.5_) exposure using the MarketScan Commercial Claims and Encounters database and the United States Environmental Protection Agency data, United States, 2017–2020. Note: *t_0_*, date of infection onset; *t_3_*, date of diagnosis for acute respiratory infection; *t*_-x_, *x* days before *t*_0_; *t*_-7_, 7 d prior to date of infection onset; *t*_28_, 28 d after initial infection onset; *t_0_* - *t_-7_*, exposure period; *t_0_* - *t_-x_*, look-back period; *t_0_* - *t_28_*, risk period. *Up to 7 d after the previous hospital discharge for hospital readmissions.

We included multiple covariates in the models including the month and year of the estimated onset of infection, child's age (in years) and biological sex, type of health insurance cover (Consumer-driven health plan, health maintenance organization, high-deductible health plan, preferred provider organization, other), region (Northeast, North Central, South, and West), population density of residential MSA (quartiles), influenza vaccination status, mean dew point temperature, and mean temperature. Month and year of the estimated onset of infection, child's age and biological sex, type of health insurance cover, and region were derived from the initial health encounter record. Population density of residential MSAs were obtained from the 2021 American Community Survey [19] and each MSA was categorized by population density into quartiles from lowest (quartile 1) to highest (quartile 4). Children with no vaccination record and children who had a vaccination record ≤2 weeks prior to the estimated date of onset of infection (insufficient time to produce protective level of antibodies) were considered unvaccinated. Children who received an influenza vaccination >2 weeks from the estimated date of onset of infection during the current influenza season were considered vaccinated. Daily meteorological data were obtained from the Parameter-elevation Regressions on Independent Slopes Model Climate Group. We calculated the average mean temperature and mean dew point temperature in the 7-day window prior to the estimated date of onset of infection based on the residential MSA [20]. Using a lookback period from the estimated date of onset of infection, we extracted information on influenza vaccination (Figure 1). For hospital readmission, we considered 7 days past the previous hospital discharge (ie, t_28_* in Figure 1).

### Statistical Analyses

We used log-binomial distributed generalized linear models for binary outcomes and Poisson distributed generalized linear models for count outcomes; we clustered within children to account for intragroup correlation of outcomes within children who contributed more than one ARI episode. Effects of PM_2.5_ were estimated using risk ratio for binary outcomes and length of hospital stay (in days) for discrete continuous (count) outcomes with 95% confidence intervals (95% CI) per interquartile range (IQR; 75th percentile-25th percentile) increase in short-term (lag 0-7 days) PM_2.5_ exposure. To evaluate the potential impact of sociodemographic factors, subgroup analyses were planned a priori and performed for potential effect modifiers, including by age group, influenza vaccination status, and MSA-level population density; additionally, we tested for effect modification to explore potential modifying impacts on the association between short-term PM_2.5_ exposure and the outcomes. In sensitivity analysis, we examined whether restriction to children who were continuously enrolled in private health insurance for an entire year prior to the date of onset of infection impacted the observed associations. In additional sensitivity analyses to evaluate the possible influence of selection bias, we further identified children with an onset of infection any time during the year and identified the cohort using different principal and/or additional diagnosis fields, including: (1) ARI (principal and/or additional diagnosis field), (2) influenza (principal diagnosis field), and (3) influenza (principal and/or additional diagnosis field). All analyses were performed in Stata/SE version 17.0 (StataCorp LLC).

## RESULTS

**Figure 2.**
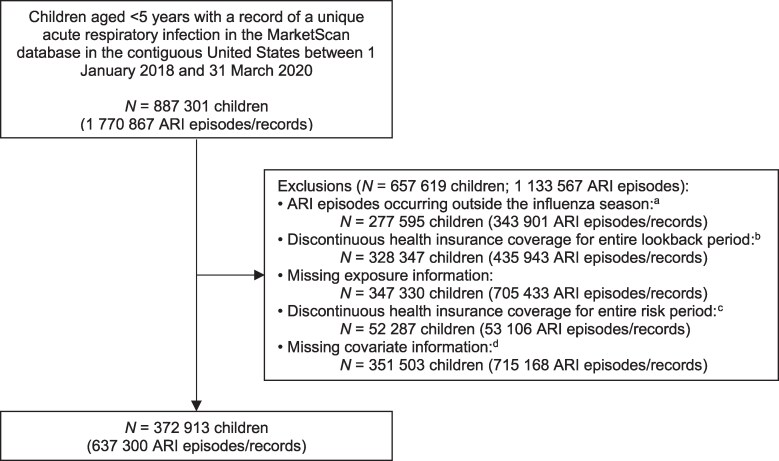
Flow diagram of study participants included in the cohort. *Acute respiratory infection obtained from ICD-10-CM codes found in the principal diagnosis field of hospital inpatient, outpatient, and emergency department records (Supplementary Table 1). ^a^Influenza season: September to April. ^b^Lookback period: previous September until the date of infection onset. ^c^Risk period: date of infection onset to 28 d after (or to 7 d after the previous hospital discharge for hospital readmissions). ^d^Covariates: month and year of infection onset, child's age and biological sex, type of health insurance cover, region, population density of residential metropolitan statistical area, mean dew point temperature, and mean temperature.

Of the 1 354 831 children identified in the MarketScan database, 916 954 (67.7%) experienced at least 1 ARI episode between January 2018 and March 2020, and with a cumulative incidence of 676.8 cases per 1000 children. After exclusions, 1 770 867 unique ARI episodes among 887 301 children aged <5 years were identified in 298 MSAs across the contiguous 48 US states, of which 637 300 unique ARI episodes were included in the final analysis; 1 133 567 (64.0%) ARI episodes were excluded, predominantly because of missing PM measurements and/or covariate information (Figure 2). ARI episodes were evenly distributed across age groups and tended to peak in January (124 707; 19.6%). Approximately half of the children were male (335 046; 52.6%), were vaccinated against influenza (350 939; 55.1%), had access to a preferred provider organization (310 720; 48.8%), resided in the southern region (346 968; 54.4%), and resided in the highest density MSA (346 279; 54.3%) (Table 1).

**Table 1.** Cohort Characteristics Stratified by PM_2.5_ Quintiles (N = 637 300 ARI Episodes)

Characteristics	All N = 637 300 (0.13–188.95 µg/m^3^)	Quintile 1 N = 82 230 (0.13–5.19 µg/m^3^)	Quintile 2 N = 125 439 (5.19–6.49 µg/m^3^)	Quintile 3 N = 142 105 (6.49–7.69 µg/m^3^)	Quintile 4 N = 148 833 (7.69–9.42 µg/m^3^)	Quintile 5 N = 138 693 (9.42–188.95 µg/m^3^)
*Age*						
< 1 y	105 460 (16.6)	13 405 (16.3)	20 882 (16.7)	23 340 (16.4)	24 174 (16.2)	23 659 (17.1)
1 to <2 y	148 668 (23.3)	19 647 (23.9)	29 107 (23.2)	32 957 (23.2)	34 630 (23.3)	32 327 (23.3)
2 to <3 y	131 648 (20.7)	16 857 (20.5)	25 837 (20.6)	29 537 (20.8)	30 964 (20.8)	28 453 (20.5)
3 to <4 y	126 246 (19.8)	16 123 (19.6)	24 927 (19.9)	28 250 (19.9)	29 602 (19.9)	27 344 (19.7)
4 to <5 y	125 278 (19.7)	16 198 (19.7)	24 686 (19.7)	28 021 (19.7)	29 463 (19.8)	26 910 (19.4)
*Biological sex*						
Female	302 254 (47.4)	38 869 (47.3)	59 427 (47.4)	67 566 (47.6)	70 604 (47.4)	65 788 (47.4)
Male	335 046 (52.6)	43 361 (52.7)	66 012 (52.6)	74 539 (52.5)	78 229 (52.6)	72 905 (52.6)
*Influenza vaccination status*						
No	286 361 (44.9)	38 334 (46.6)	57 511 (45.9)	64 557 (45.4)	65 425 (44.0)	60 534 (43.7)
Yes	350 939 (55.1)	43 896 (53.4)	67 928 (54.2)	77 548 (54.6)	83 408 (56.0)	78 159 (56.4)
*Type of health insurance cover*						
Consumer-driven health plan	83 622 (13.1)	10 068 (12.2)	15 864 (12.7)	17 788 (12.5)	19 906 (13.4)	19 996 (14.4)
Health maintenance organization	68 139 (10.7)	7350 (8.9)	13 144 (10.5)	16 061 (11.3)	16 207 (10.9)	15 377 (11.1)
High-deductible health plan	112 593 (17.7)	15 262 (18.6)	21 600 (17.2)	24 031 (16.9)	26 036 (17.5)	25 664 (18.5)
Preferred provider organization	310 720 (48.8)	41 379 (50.3)	62 461 (49.8)	70 098 (49.3)	72 105 (48.5)	64 677 (46.6)
Other	62 226 (9.8)	8171 (9.9)	12 370 (9.9)	14 127 (9.9)	14 579 (9.8)	12 979 (9.4)
*Region*						
North Central	143 546 (22.5)	17 660 (21.5)	24 560 (19.6)	25 961 (18.2)	32 817 (22.1)	42 648 (30.8)
Northeast	46 978 (7.4)	8394 (10.2)	10 667 (8.5)	9493 (6.7)	9308 (6.3)	9116 (6.6)
South	346 968 (54.4)	31 195 (37.9)	74 014 (59.0)	91 274 (64.2)	88 667 (59.6)	61 818 (44.6)
West	99 808 (15.7)	24 981 (30.4)	16 198 (12.9)	15 477 (10.9)	18 041 (12.1)	25 111 (18.1)
*Population density of residential metropolitan statistical area (quartiles)*						
Quartile 1 (lowest)	31 665 (5.0)	10 072 (12.3)	5172 (4.1)	4944 (3.5)	5357 (3.6)	6120 (4.4)
Quartile 2 (second lowest)	68 888 (10.8)	14 341 (17.4)	13 593 (10.8)	12 665 (8.9)	12 921 (8.7)	15 368 (11.1)
Quartile 3 (second highest)	190 468 (29.9)	24 376 (29.6)	39 698 (31.7)	42 420 (29.9)	45 542 (30.6)	38 432 (27.7)
Quartile 4 (highest)	346 279 (54.3)	33 441 (40.7)	66 976 (53.4)	82 076 (57.8)	85 013 (57.1)	78 773 (56.8)
*Month of infection onset*						
January	124 707 (19.6)	12 585 (15.3)	22 046 (17.6)	26 131 (18.4)	32 748 (22.0)	31 197 (22.5)
February	107 798 (16.9)	12 408 (15.1)	21 065 (16.8)	25 711 (18.1)	26 997 (18.1)	21 617 (15.6)
March	83 063 (13.0)	13 622 (16.6)	14 759 (11.8)	17 348 (12.2)	19 606 (13.2)	17 728 (12.8)
April	18 312 (7.6)	7766 (9.4)	11 881 (9.5)	11 244 (7.9)	11 978 (8.1)	5443 (3.9)
September	27 459 (4.3)	4347 (5.3)	5879 (4.7)	5667 (4.0)	5880 (4.0)	5686 (4.1)
October	73 085 (11.5)	17 246 (21.0)	21 563 (17.2)	18 613 (13.1)	9252 (6.2)	6411 (4.6)
November	89 088 (14.0)	7698 (9.4)	15 809 (12.6)	19 443 (13.7)	21 655 (14.6)	24 483 (17.7)
December	83 788 (13.2)	6558 (8.0)	12 437 (9.9)	17 948 (12.6)	20 717 (13.9)	26 128 (18.8)
*Year of infection onset*						
2018	278 712 (43.7)	36 151 (44.0)	56 240 (44.8)	63 486 (44.7)	64 060 (43.0)	58 775 (42.4)
2019	269 879 (42.4)	33 332 (40.5)	49 148 (39.2)	56 530 (39.8)	62 546 (42.0)	68 323 (49.3)
2020	88 709 (13.9)	12 747 (15.5)	20 051 (16.0)	22 089 (15.5)	22 227 (14.9)	11 595 (8.4)

Data are presented as frequency (percentage).

Of the 637 300 ARI episodes contributed by 372 913 children, 222 436 (59.6%), 86 473 (23.2%), 36 101 (9.7%), and 27 903 (7.5%) children experienced 1, 2, 3, and 4 or more unique ARI episodes during the study period, respectively. There were 3715 (0.58%), 6194 (0.97%), 83 (1.43%), 1827 (0.29%), and 184 (0.03%) episodes of antiviral prescription claims, hospital admission, hospital readmission, ICU admissions, and mechanical ventilation, respectively. The IQR of PM_2.5_ exposure was 3.34 (range: 0.13-188.95) µg/m^3^.

There were 3715 (0.58%) ARI episodes among 3695 children that resulted in a prescription claim for antiviral medication. In the main analysis, we observed an 11% increase in the risk of antiviral prescription claims per IQR increase in PM_2.5_ exposure (aRR, 1.11; 95% CI, 1.07-1.15) (Table 2). This association was consistently observed when stratifying by age (aged <2 years: adjusted risk ratio [aRR], 1.14; 95% CI, 1.10-1.18; aged 2 to <5 years: 1.09; 95% CI, 1.04-1.15) (Supplementary Table 2), biological sex (female: aRR, 1.14; 95% CI, 1.09-1.20; male, 1.08; 95% CI, 1.03-1.14) (Supplementary Table 3), influenza vaccination status (unvaccinated: aRR, 1.07; 95% CI, 1.004-1.14; vaccinated: aRR, 1.15; 95% CI, 1.12-1.15) (Supplementary Table 4), and MSA-level population density (highest: aRR, 1.15; 95% CI, 1.12-1.18) (Supplementary Table 5). This association also persisted in sensitivity analyses among children who had experienced an ARI any time during the year (aRR, 1.09; 95% CI, 1.05-1.14) (Supplementary Table 6) or when considering different methods of diagnosis for an infection (ARI diagnosis in the principal and/or additional diagnosis field: aRR, 1.12; 95% CI, 1.09-1.16; influenza diagnosis in the principal diagnosis field: aRR, 1.10; 95% CI, 1.05-1.15; influenza diagnosis in the principal and/or additional diagnosis field: aRR, 1.11; 95% CI, 1.07-1.15) (Supplementary Table 7). We observed evidence of effect modification by influenza vaccination status for PM_2.5_ and risk of antiviral prescription claims (*p*_interaction_ = 0.019).

**Table 2.** Risk Ratios and Length of Stay in Hospital of Severe Acute Respiratory Infection Outcomes Associated With an Interquartile Range (3.34 µg/m^3^) Increase in PM_2.5_ Exposure Among Young Children Aged <5 Years

	Antiviral Prescription Claim	Hospital Admission	Hospital Readmission	ICU Admission	Mechanical Ventilation	Length of Stay in Hospital (hospital-admitted children)^a^	Length of Stay in Hospital (ICU-admitted children)^a^
N (children)	372 913	372 913	5413	372 913	372 913	5686	1696
N (episodes)	637 300	637 300	5819	637 300	637 300	6194	1827
n (children with outcome)	3695 (0.99)	5686 (1.52)	81 (1.50)	1696 (0.45)	63 (0.02)	…	…
n (outcome)	3715 (0.58)	6194 (0.97)	83 (1.43)	1827 (0.29)	184 (0.03)	…	…
RR (95% CI)	**1.08 (1.07-1.09)**	**1.03 (1.01-1.05)**	0.98 (0.81-1.18)	**1.06 (1.02-1.09)**	0.96 (0.76-1.22)	**0.040 (0.007-0.072)**	0.042 (−0.013 to 0.096)
aRR (95% CI)^b^	**1.11 (1.07-1.15)**	1.00 (0.97-1.02)	0.97 (0.79-1.21)	1.02 (0.98-1.06)	0.97 (0.77-1.22)	**0.041 (0.006-0.075)**	0.049 (−0.009 to 0.107)

Abbreviations: aRR, adjusted risk ratio; CI, confidence interval; ICU, intensive care unit; RR, unadjusted risk ratio.

Bold values indicate statistically significant associations (ie, 95% CI does not cross 1).

^a^Length of stay in hospital (in days).

^b^Risk ratios and length of stay adjusted for the month and year of infection onset, child's age and biological sex, influenza vaccination status, type of health insurance cover, region, population density of residential metropolitan statistical area, mean dew point temperature, and mean temperature.

Of the 637 300 ARI episodes included in the study, 6194 (0.97%) resulted in a hospital admission (Table 2). In the main analysis, we observed no association between short-term PM_2.5_ exposure and hospital admissions (Table 2). In sensitivity analyses, there was a 6% decrease in the risk of hospital admissions among children with 1 year of continuous health insurance coverage (aRR, 0.94; 95% CI, 0.89-0.98) (Supplementary Table 6). No associations were observed when stratifying by age (Supplementary Table 2), biological sex (Supplementary Table 3), influenza vaccination status (Supplementary Table 4), or MSA-level population density (Supplementary Table 5). There were effect modifications by age group and influenza vaccination status on the association between short-term PM_2.5_ exposure and the risk of hospital admission (*p*_interaction_ = 0.009 and 0.040, respectively).

Of the 6194 hospital admissions for an ARI, 5827 (94.1%) of the total ARI admissions resulted in a length of stay of ≤ 7 days, of which 8 (0.1%) episodes were excluded due to discontinuous health insurance coverage for the risk period for hospital readmissions. Of 5819 episodes among 5413 children admitted to hospital with an ARI; 83 (1.43%) resulted in a readmission within 7 days of the previous hospital discharge (Table 2). In the main analysis, there was no association between short-term PM_2.5_ exposure and hospital readmission (Table 2). In subgroup analyses, we observed a 10% increase in the risk of hospital readmission among female children per IQR increase in PM_2.5_ exposure (aRR, 1.10; 95% CI, 1.005-1.20) (Supplementary Table 3). No associations were observed among male children (Supplementary Table 3) or when stratifying by age (Supplementary Table 2), influenza vaccination status (Supplementary Table 4), or MSA-level population density (Supplementary Table 5). There was no effect modification by age group (Supplementary Table 2), biological sex (Supplementary Table 3), influenza vaccination status (Supplementary Table 4), or MSA-level population density (Supplementary Table 5) for any of the models.

A total of 1827 (0.29%) ICU admissions were identified among 1696 children aged <5 years. We observed no association between short-term PM_2.5_ exposure and the risk of ICU admission (**Table 2**). In subgroup analyses, we observed a 6% and 7% increased risk of ICU admission among unvaccinated children (aRR, 1.06; 95% CI, 1.02-1.10) (Supplementary Table 4) and children residing in the second lowest density MSAs (aRR, 1.07; 95% CI, 1.03-1.10) (Supplementary Table 5) per IQR increase in PM_2.5_ exposure, respectively. There were no associations observed among vaccinated children (Supplementary Table 4), children residing in other population-dense MSAs (Supplementary Table 5), or when stratifying by age (Supplementary Table 2) or biological sex (Supplementary Table 3). There was evidence of effect modification by influenza vaccination status (*p*_interaction_ = 0.006) for short-term PM_2.5_ exposure and ICU admission.

In total, there were 184 (0.03%) ARI episodes among 63 children that resulted in the requirement for mechanical ventilation (Table 2). There was no association between short-term PM_2.5_ exposure and mechanical ventilation (Table 2). When stratifying by MSA-level population density, we observed a significant association with mechanical ventilation among children residing in the highest density MSAs (aRR, 1.09; 95% CI, 1.01-1.18) (Supplementary Table 5). This association was not observed among children residing in other population-dense MSAs (Supplementary Table 5), or when stratifying by age (Supplementary Table 2), biological sex (Supplementary Table 3), or influenza vaccination status (Supplementary Table 4). No effect modification by age group (Supplementary Table 2), biological sex (Supplementary Table 3), influenza vaccination status (Supplementary Table 4), or MSA-level population density (Supplementary Table 5) was observed for short-term PM_2.5_ exposure and mechanical ventilation.

The median length of stay in hospital among hospital-admitted children was 2 days (IQR, 2 days). In the main analysis, hospital-admitted children had a longer length of stay in hospital per IQR increase in PM_2.5_ exposure (adjusted, 0.041 days; 95% CI, 0.006-0.075) (Supplementary Table 2). This association was consistently observed male children (adjusted, 0.059; 95% CI, 0.008-0.110) (Supplementary Table 3), unvaccinated children (adjusted, 0.065; 95% CI, 0.007-0.124) (Supplementary Table 4), children who resided in the second highest density MSAs (adjusted, 0.087 days; 95% CI, 0.011-0.162) (Supplementary Table 5). This association also persisted in sensitivity analyses among hospital-admitted children with 1 year of continuous health insurance coverage (adjusted, 0.125; 95% CI, 0.046-0.204) (Supplementary Table 6), diagnosed with an ARI any time during the year (adjusted, 0.036; 95% CI, 0.003-0.068) (Supplementary Table 6), and who were diagnosed with influenza in the principal diagnosis field (adjusted, 0.208; 95% CI, 0.015-0.400) (Supplementary Table 7). There was no association among female children (Supplementary Table 3), vaccinated children (Supplementary Table 4), children residing in other population-dense MSAs (Supplementary Table 5), or when stratifying by age (Supplementary Table 2). No effect modification by age group (Supplementary Table 2), biological sex (Supplementary Table 3), influenza vaccination status (Supplementary Table 4), or MSA-level population density (Supplementary Table 5) was observed for length of stay in hospital among hospital-admitted children.

The median length of stay in hospital among ICU-admitted children was 3 days (IQR, 3 days), respectively. There was no association between short-term PM_2.5_ exposure and length of stay in hospital among ICU-admitted children (Table 2). In sensitivity analyses, we observed an association between short-term PM_2.5_ exposure and length of stay in hospital among ICU-admitted children with 1 year of continuous health insurance coverage (adjusted, 0.151; 95% CI, 0.065-0.238) (Supplementary Table 6), diagnosed with influenza in the principal diagnosis field (adjusted, 0.331; 95% CI, 0.114-0.548) or principal and/or additional diagnosis field (adjusted, 0.290; 95% CI, 0.068-0.512) (Supplementary Table 7). No associations were observed when stratifying by age (Supplementary Table 2), biological sex (Supplementary Table 3), influenza vaccination status (Supplementary Table 4), or MSA-level population density (Supplementary Table 5). We found no evidence of effect modification by age group (Supplementary Table 2), biological sex (Supplementary Table 3), influenza vaccination status (Supplementary Table 4), or MSA-level population density (Supplementary Table 5) for length of stay in hospital among ICU-admitted children.

## DISCUSSION

Using private health insurance claims data, we conducted a claims-based cohort study to estimate the association between short-term PM_2.5_ exposure and the risk of severe ARI outcomes among young children aged <5 years. We identified a significant positive association between short-term PM_2.5_ exposure and the presence of a prescription claim record for antiviral medication. These results were consistent when stratifying by children's age, biological sex, influenza vaccination status, when examining children who had experienced an ARI any time during the year, and when considering different methods of diagnosis for an infection. In addition, we found a found a higher risk of ICU admission and a longer length of stay in hospital among children not vaccinated against influenza. These associations were not observed among children who were vaccinated against influenza, which may suggest that exposure to vaccines may lead to milder respiratory illness [21, 22]. We also found that female children had a higher risk of being readmitted to hospital, which was not observed among male children, and we observed a higher risk of mechanical ventilation among children residing in the highest population-dense MSAs. To our knowledge, no other study has reported our significant associations between exposure to short-term PM_2.5_ and the presence of a prescription claim record for antiviral medication, hospital readmission, ICU admission, mechanical ventilation, and length of stay in hospital. These findings may suggest that short-term PM_2.5_ exposure may contribute to severe ARI-associated illness among young children, and that influenza vaccination may modify the risk of severe ARI-associated outcomes.

To date, most studies evaluating the impact of air pollution have focused on risk of respiratory infection (eg, ARIs, influenza, pneumonia) diagnoses. However, severe ARI outcomes have been infrequently examined, particularly among young children. A US study by Strosnider et al [23]. examined 39 975 411 respiratory emergency department visits in 894 counties from 17 states, which 16 096 443 occurred among children aged <19 years. This study observed an increased risk of emergency department visits for all-cause respiratory disease (aRR, 1.024; 95% CI, 1.019-1.029), ARI (aRR, 1.021; 95% CI, 1.016-1.027), and asthma (aRR, 1.034; 95% CI, 1.021-1.047) per 10 µg/m^3^ increase in exposure to 24-hour average PM_2.5_. A similar study conducted in China by Lei et al [11] identified 1 399 955 hospital admissions, of which 739 687 occurred among children aged ≤17 years reported an increased risk of hospital admissions for all-cause respiratory disease per 34.5 µg/m^3^ increase in lag 0–4 day average PM_2.5_ exposure (aRR, 1.022; 95% CI, 1.015-1.029) and per 26.0 µg/m^3^ increase in lag 0–4 day average PM_2.5-10_ exposure (aRR, 1.025; 95% CI, 1.018-1.031).

Previous studies have proposed biological mechanisms to explain the observed association between PM_2.5_ and respiratory infections. Recent studies suggest that respiratory droplets can attach to smaller PM sizes (ie, PM_2.5_) and therefore be suspended in the air for longer periods and consequently increase the opportunity and transmissibility of respiratory infection [24]. PM particles may have the potential to penetrate deeper into the lower respiratory tract, remain in the airways for longer, and further exacerbating the pathogenesis of respiratory diseases [25]. Studies have also suggested that PM_2.5_ weakens the body's immunological defenses, leading to an increased susceptibility to infection and/or impairs the immune responses to infection, therefore, leading to more severe respiratory disease [7].

The main strengths of our study include the availability of a large national claims-based cohort with detailed information on healthcare utilization through the integration of inpatient and outpatient care, emergency care services, as well as outpatient pharmaceutical data. Additionally, instead of relying on prescription practices as a proxy for medication usage, the MarketScan database records prescriptions that have been filled and claimed for the covered individual.

However, our study also has several limitations. Although the MarketScan database allows the examination of a large population-based cohort throughout the contiguous United States, the database is limited by the absence of those who do not have private health insurance provided by an employer and those residing in rural areas. This could limit the generalizability of our results from other groups due to socioeconomic factors and therefore cannot be generalized to all US children. We were not able to account for the potential confounding effects of race/ethnicity and socioeconomic status. Since we used the average ambient PM_2.5_ levels at the child's residential MSA as surrogates for individual exposure, we cannot rule out the possibility of exposure misclassification from individual activity patterns (ie, outdoor mobility patterns) and/or heterogeneity of PM_2.5_ concentrations within an MSA. PM_2.5_ is a complex mixture with a wide range of sources (eg, vehicles, agriculture, industry, wildfires). Its chemical structure can vary widely by location and season. Previous research has shown that the health burden of PM_2.5_ differs based on chemical structure and source [26]. Additionally, the distribution of PM_2.5_ is highly skewed (5th quintile: 9.42-188.95 µg/m^3^). Based on monitoring data, which are disproportionately located in urban settings, the mean PM_2.5_ level in the United States for 2018 through 2020 was 7.9 µg/m^3^, with the highest concentration in the West (10.6 µg/m^3^) and lowest in the Northern Rockies and Plains (5.9 µg/m^3^). The current health-based annual standard for PM_2.5_ is 9 µg/m^3^ [27], although >20 million people in the United States reside in areas that do not comply with the standard. Future work is needed to investigate the severity of acute respiratory infections in children impacted by PM_2.5_ exposure at various concentrations, including extreme levels of PM_2.5_, as well as the impact of different chemical structures, sources, and air pollution mixtures. We did not have access to laboratory data; the identification of the study cohort (children with an ARI) relied on diagnostic codes that may have limited specificity. However, studies have reported high specificity of ARIs (ie, influenza, respiratory syncytial virus) detected through diagnostic coding compared to laboratory confirmation [7].

## CONCLUSION

We found empirical evidence that short-term PM_2.5_ exposure may contribute to severe ARI outcomes after ARI infection among children aged <5 years. Few studies have evaluated the association between PM_2.5_ exposure and severe ARI outcomes in children. As respiratory illnesses and ambient PM_2.5_ concentrations continue to increase over time, it will become increasingly important to understand the impact PM_2.5_ has on respiratory health, particularly in children due to the disproportionate risk for ARI hospitalization compared to their older counterparts [28]. Further research is also needed to distinguish whether wildfire smoke during pregnancy has different risks on children based on socioeconomic factors and access to healthcare. Although further research is needed, our results support the need for air quality guidelines and policies to protect health and welfare, the need to regulate PM emissions and maintain air quality standards and suggest the need for additional studies with more generalizable cohorts.

## Supplementary Material

ofaf442_Supplementary_Data

## Supplementary Data

Supplementary materials are available at *Open Forum Infectious Diseases* online. Consisting of data provided by the authors to benefit the reader, the posted materials are not copyedited and are the sole responsibility of the authors, so questions or comments should be addressed to the corresponding author.
