# Supplementary material for: Association Between Fine Particulate Matter (PM2.5) and Severity of Acute Respiratory Infections Among Young US Children in the Major Cities in the United States: A Claims-based Cohort Study
Source: Open Forum Infect Dis. 2025 Oct 10;12(10):ofaf442. doi: 10.1093/ofid/ofaf442 (PMC12548786; doi:10.1093/ofid/ofaf442)
Supplement: ofaf442_Supplementary_Data [file ofaf442_supplementary_data.docx]

**S1 Table**. Diagnostic, procedural, and pharmaceutical codes for all-cause acute respiratory infections, intensive care unit admissions, mechanical ventilation, and antiviral drug prescription claims.

| Variable | Code | |
| --- | --- | --- |
| *Outcome:* |  | |
| All-cause acute respiratory infection | ICD-10-CM codes:  A37, B34, B97.4, B97.8, J00, J01, J02, J03, J04, J06, J09, J10, J11, J12, J13, J14, J15, J16, J17, J18, J20, J21, J22, , J39.8, J39.9, J40, J80, J81, J90, J96.0, J96.2, J96.90, J98.09, J98.11, J98.19, J98.4, J98.8, J98.9, O98.51, O98.52, O98.53, O98.81, O99.51 | |
| Intensive care unit admission | CPT codes:  99291-99292, 99468-99472, 99475-99479, 99480  HCPCS codes:  G9853 | |
| Mechanical ventilation | ICD-10-CM codes:  Z99.0  CPT codes:  4168F, 94002, 94003, 94004, 94662, 94656, 94657, 99504  HCPCS codes:  A4483 | |
| *Antiviral prescription:* |  |  |
| TamiFlu (brand name) | NDC codes:  0004-0800-07, 0004-0800-08, 0004-0800-85, 0004-0801-85, 0004-0802-85, 0004-0822-05, 50090-1244-0, 70518-2042-0, 50090-0662-0 | |
| Oseltamivir (trade name) | NDC codes:  0093-8180-64, 16714-817-01, 16714-818-01, 16714-819-01, 27241-139-09, 31722-108-60, 31722-630-31, 31722-631-31, 31722-632-31, 42291-567-60, 42291-664-10, 42291-666-10, 46708-413-10, 46708-414-10, 46708-415-10, 47781-384-26, 47781-468-13, 47781-469-13, 47781-470-13, 50090-3710-0, 50090-3718-0, 50090-4159-0, 50090-4188-0, 50090-4613-0, 50090-4650-0, 50090-4783-0, 50090-4890-0, 50090-5631-0, 50090-6099-0, 50090-6239-0, 53002-1387-1, 53002-1710-1, 53002-3710-1, 53002-4387-1, 53002-4710-1, 53002-5387-1, 55700-793-10, 55700-798-60, 60219-1264-1, 60219-1265-1, 60219-1266-1, 62332-413-10, 62332-414-10, 62332-415-10, 64380-797-01, 64380-798-01, 64380-799-01, 64380-799-06, 64380-799-07, 64380-879-75, 68071-2528-1, 68071-2565-1, 68071-2612-1, 68071-2754-1, 68071-4733-6, 68071-4743-6, 68180-675-10, 68180-675-11, 68180-676-10, 68180-676-11, 68180-677-10, 68180-677-11, 68180-678-01, 68788-7600-6, 68788-8122-1, 68788-8123-6, 69238-1264-1, 69238-1265-1, 69238-1266-1, 69238-1273-6, 69315-174-60, 69539-109-33, 69539-110-33, 69539-111-33, 70518-2397-0, 70518-3247-0, 70710-1008-2, 70710-1009-2, 70710-1010-2, 70710-1165-6, 70771-1598-6, 70771-1710-2, 70771-1711-2, 70771-1712-2, 71205-206-10, 71205-206-30, 71205-206-60, 71205-206-90, 71205-359-10, 71205-360-10, 71205-397-10, 71205-489-60, 71205-617-10, 71205-680-10, 72205-042-11, 72205-043-11, 72205-044-11, 72205-060-78, 76420-289-10, 76420-290-10, 76420-291-10 | |
| *Vaccine:* |  | |
| Influenza vaccine | NDC codes:  19515089101, 19515089111, 19515089302, 19515089307, 19515089441, 19515089452, 19515089801, 19515089811, 19515090301, 19515090311, 19515090841, 19515090852, 19515089601, 19515089611, 19515091241, 19515091252, 33332001401, 33332001402, 33332011410, 33332011411, 33332011510, 33332011511, 33332001501, 33332001502, 33332031601, 33332031602, 33332011611, 33332011610, 33332001601, 33332001602, 33332031701, 33332031702, 33332041710, 33332041711, 33332011710, 33332011711, 33332001701, 33332001702, 58160088141, 58160088152, 58160090141, 58160090152, 58160090341, 58160090352, 58160090541, 58160090552, 58160090741, 58160090752, 62577061301, 62577061311, 62577061401, 62577061411, 70461020001, 70461020011, 70461020101, 70461020111, 70461030110, 70461030112, 70461031803, 70461031804, 70461041810, 70461041811, 66019030101, 66019030110, 66019030201, 66019030210, 66019030301, 66019030310, 66019030401, 66019030410, 66521000001, 66521000011, 70461000101, 70461000111, 70461000201, 70461000211, 42874001401, 42874001410, 42874001501, 42874001510, 42874001601, 42874001610, 42874001701, 42874001710, 42874011701, 42874011710, 66521011702, 66521011710, 66521011711, 66521011712, 66521011802, 66521011810, 66521011811, 66521011812, 70461011902, 70461011910, 70461011911, 70461011912, 70461012002, 70461012010, 70461012011, 70461012012, 49281039415, 49281039478, 49281039565, 49281039588, 49281062115, 49281062178, 49281001450, 49281001488, 49281041410, 49281041450, 49281041458, 49281041488, 49281051400, 49281051425, 49281070840, 49281070848, 49281070948, 49281070955, 49281041510, 49281041550, 49281041558, 49281041588, 49281051500, 49281051525, 49281062315, 49281051500, 49281051525, 49281062378, 49281039615, 49281039678, 49281039765, 49281039788, 49281039965, 49281039988, 49281040165, 49281040188, 49281040365, 49281040388, 49281041610, 49281041650, 49281041658, 49281041688, 49281051600, 49281051625, 49281062515, 49281062578, 49281062515, 49281062578, 49281071040, 49281071048, 49281041710, 49281041750, 49281041758, 49281041788, 49281051700, 49281051725, 49281062715, 49281062778, 49281071240, 49281071248  CPT codes:  90630, 90653, 90654, 90655, 90656, 90657, 90658, 90659, 90660, 90661, 90662, 90663, 90664, 90666, 90667, 90668, 90672, 90673, 90674, 90682, 90685, 90686, 90687, 90688, 90689, 90756, 90686, 90694  HCPCS codes:  G0008, G8108, G8482, G8483, G8636, G8639, G9141, G9142, Q2033, Q2034, Q2035, Q2036, Q2037, Q2038, Q2039 | |
| Abbreviations: CPT, Current Procedural Terminology; HCPCS, Healthcare Common Procedure Coding System; ICD-10-CM, International Statistical Classification of Diseases and Related Health Problems, Tenth Revision, Clinical Modification; NDC, National Drug Code. | | |

**S2 Table**. Risk ratios and length of stay in hospital for severe acute respiratory infection outcomes associated with an interquartile range (3.34 µg/m^3^) increase in PM_2.5_ exposure among young children aged <5 years, by age group.

|  | **Antiviral prescription claim** | **Hospital admission** | **Hospital readmission** | **ICU admission** | **Mechanical ventilation** | **Length of stay in hospital (hospital-admitted children)^a^** | **Length of stay in hospital (ICU-admitted children)^a^** |
| --- | --- | --- | --- | --- | --- | --- | --- |
| *Children aged <2 years* | | | | | | | |
| **N (children)** | 163,347 | 163,347 | 3,580 | 163,347 | 163,347 | 3,756 | 1,194 |
| **N (episodes)** | 254,128 | 254,128 | 3,804 | 254,128 | 254,128 | 4,037 | 1,272 |
| **n (children with outcome)** | 1,279 (0.78) | 3,756 (2.30) | 52 (1.45) | 1,194 (0.73) | 39 (0.02) |  |  |
| **n (outcome)** | 1,281 (0.50) | 4,037 (1.59) | 52 (1.37) | 1,272 (0.50) | 97 (0.04) |  |  |
| **RR (95% CI)** | **1.08 (1.07 to 1.10)** | **1.05 (1.03 to 1.07)** | 1.02 (0.84 to 1.24) | **1.05 (1.03 to 1.08)** | 0.96 (0.74 to 1.25) | 0.026 (-0.007 to 0.059) | 0.024 (-0.049 to 0.098) |
| **aRR (95% CI)*** | **1.14 (1.10 to 1.18)** | 1.02 (0.99 to 1.04) | 1.05 (0.84 to 1.32) | 1.02 (0.97 to 1.06) | 1.02 (0.83 to 1.24) | 0.024 (-0.011 to 0.060) | 0.031 (-0.049 to 0.111) |
| ***p*_interaction_** | [Reference] | [Reference] | [Reference] | [Reference] | [Reference] | [Reference] | [Reference] |
| *Children aged 2 to <5 years* | | | | | | | |
| **N (children)** | 241,898 | 241,898 | 1,885 | 241,898 | 241,898 | 1,993 | 513 |
| **N (episodes)** | 383,172 | 383,172 | 2,015 | 383,172 | 383,172 | 2,157 | 555 |
| **n (children with outcome)** | 2,421 (1.00) | 1,993 (0.82) | 29 (1.54) | 513 (0.21) | 32 (0.01) |  |  |
| **n (outcome)** | 2,434 (0.64) | 2,157 (0.56) | 31 (1.54) | 555 (0.14) | 87 (0.02) |  |  |
| **RR (95% CI)** | **1.07 (1.06 to 1.09)** | 0.98 (0.92 to 1.03) | 0.9 (0.65 to 1.24) | 1.05 (0.97 to 1.13) | 0.96 (0.64 to 1.44) | 0.064 (-0.002 to 0.13) | 0.053 (-0.021 to 0.127) |
| **aRR (95% CI)*** | **1.09 (1.04 to 1.15)** | 0.95 (0.91 to 1.003) | 0.85 (0.59 to 1.22) | 1.02 (0.94 to 1.11) | 0.89 (0.60 to 1.33) | 0.067 (-0.0003 to 0.133) | 0.07 (-0.010 to 0.149) |
| ***p*_interaction_^b^** | 0.195 | **0.009** | 0.547 | 0.827 | 0.980 | 0.336 | 0.657 |
| Abbreviations: aRR, adjusted risk ratio; CI, confidence interval; ICU, intensive care unit; RR, unadjusted risk ratio.  Bold values indicate statistically significant associations (i.e., 95% CI does not cross 1).  * Risk ratios and length of stay adjusted for the month and year of infection onset, child's biological sex, influenza vaccination status, type of health insurance cover, region, population density of residential metropolitan statistical area, mean dew point temperature, and mean temperature.  ^a^ Length of stay in hospital (in days).  ^b^ Referent: Children aged <2 years. | | | | | | | |

**S3 Table**. Risk ratios and length of stay in hospital for severe acute respiratory infection outcomes associated with an interquartile range (3.34 µg/m^3^) increase in PM_2.5_ exposure among young children aged <5 years, by child’s biological sex.

|  | **Antiviral prescription claim** | **Hospital admission** | **Hospital readmission** | **ICU admission** | **Mechanical ventilation** | **Length of stay in hospital (hospital-admitted children)^a^** | **Length of stay in hospital (ICU-admitted children)^a^** |
| --- | --- | --- | --- | --- | --- | --- | --- |
| *Female children* | | | | | | | |
| **N (children)** | 178,701 | 178,701 | 2,297 | 178,701 | 178,701 | 2,430 | 739 |
| **N (episodes)** | 302,254 | 302,254 | 2,486 | 302,254 | 302,254 | 2,665 | 791 |
| **n (children with outcome)** | 1,748 (0.98) | 2,430 (1.36) | 29 (1.26) | 739 (0.41) | 34 (0.02) |  |  |
| **n (outcome)** | 1,758 (0.58) | 2,665 (0.88) | 31 (1.25) | 791 (0.26) | 84 (0.03) |  |  |
| **RR (95% CI)** | **1.09 (1.07 to 1.10)** | **1.05 (1.02 to 1.08)** | **1.09 (1.02 to 1.17)** | **1.08 (1.05 to 1.11)** | 0.93 (0.72 to 1.20) | 0.023 (-0.023 to 0.069) | -0.004 (-0.114 to 0.105) |
| **aRR (95% CI)*** | **1.14 (1.09 to 1.20)** | 1.01 (0.98 to 1.05) | **1.10 (1.005 to 1.20)** | 1.04 (0.99 to 1.08) | 0.85 (0.63 to 1.15) | 0.024 (-0.025 to 0.074) | 0.023 (-0.080 to 0.126) |
| ***p*_interaction_** | 0.147 | 0.241 | 0.074 | 0.159 | 0.786 | 0.345 | 0.275 |
|  | | | | | | | |
| **N (children)** | 194,212 | 194,212 | 3,116 | 194,212 | 194,212 | 3,256 | 957 |
| **N (episodes)** | 335,046 | 335,046 | 3,333 | 335,046 | 335,046 | 3,529 | 1,036 |
| **n (children with outcome)** | 1,947 (1.00) | 3,256 (1.68) | 52 (1.67) | 957 (0.49) | 29 (0.01) |  |  |
| **n (outcome)** | 1,957 (0.58) | 3,529 (1.05) | 52 (1.56) | 1,036 (0.31) | 100 (0.03) |  |  |
| **RR (95% CI)** | **1.07 (1.06 to 1.08)** | 1.02 (0.99 to 1.05) | 0.80 (0.57 to 1.13) | 1.03 (0.98 to 1.09) | 0.99 (0.69 to 1.41) | **0.058 (0.010 to 0.105)** | 0.062 (-0.003 to 0.128) |
| **aRR (95% CI)*** | **1.08 (1.03 to 1.14)** | 0.98 (0.95 to 1.02) | 0.83 (0.58 to 1.20) | 0.99 (0.93 to 1.06) | 1.04 (0.81 to 1.33) | **0.059 (0.008 to 0.110)** | 0.067 (-0.004 to 0.139) |
| ***p*_interaction_^b^** | [Reference] | [Reference] | [Reference] | [Reference] | [Reference] | [Reference] | [Reference] |
| Abbreviations: aRR, adjusted risk ratio; CI, confidence interval; ICU, intensive care unit; RR, unadjusted risk ratio.  Bold values indicate statistically significant associations (i.e., 95% CI does not cross 1).  * Risk ratios and length of stay adjusted for the month and year of infection onset, child's age, influenza vaccination status, type of health insurance cover, region, population density of residential metropolitan statistical area, mean dew point temperature, and mean temperature.  ^a^ Length of stay in hospital (in days).  ^b^ Referent: Male children. | | | | | | | |

**S4 Table**. Risk ratios and length of stay in hospital for severe acute respiratory infection outcomes associated with an interquartile range (3.34 µg/m^3^) increase in PM_2.5_ exposure among young children aged <5 years, by child's influenza vaccination status.

|  | **Antiviral prescription claim** | **Hospital admission** | **Hospital readmission** | **ICU admission** | **Mechanical ventilation** | **Length of stay in hospital (hospital-admitted children)^a^** | **Length of stay in hospital (ICU-admitted children)^a^** |
| --- | --- | --- | --- | --- | --- | --- | --- |
| *Children not vaccinated against influenza* | | | | | | | |
| **N (children)** | 199,687 | 199,687 | 2,585 | 199,687 | 199,687 | 2,740 | 785 |
| **N (episodes)** | 286,361 | 286,361 | 2,690 | 286,361 | 286,361 | 2,885 | 825 |
| **n (children with outcome)** | 1,816 (0.91) | 2,740 (1.37) | 36 (1.39) | 785 (0.39) | 31 (0.02) |  |  |
| **n (outcome)** | 1,823 (0.64) | 2,885 (1.01) | 37 (1.38) | 825 (0.29) | 57 (0.02) |  |  |
| **RR (95% CI)** | **1.07 (1.05 to 1.09)** | **1.05 (1.03 to 1.07)** | 0.78 (0.55 to 1.09) | **1.09 (1.06 to 1.12)** | 1.06 (0.88 to 1.27) | 0.055 (-0.0004 to 0.111) | 0.028 (-0.040 to 0.095) |
| **aRR (95% CI)*** | **1.07 (1.004 to 1.14)** | 1.02 (0.99 to 1.05) | 0.76 (0.52 to 1.12) | **1.06 (1.02 to 1.10)** | 1.05 (0.87 to 1.26) | **0.065 (0.007 to 0.124)** | 0.049 (-0.026 to 0.125) |
| ***p*_interaction_** | [Reference] | [Reference] | [Reference] | [Reference] | [Reference] | [Reference] | [Reference] |
| *Children vaccinated against influenza* | | | | | | | |
| **N (children)** | 214,851 | 214,851 | 2,914 | 214,851 | 214,851 | 3,044 | 937 |
| **N (episodes)** | 350,939 | 350,939 | 3,129 | 350,939 | 350,939 | 3,309 | 1,002 |
| **n (children with outcome)** | 1,883 (0.88) | 3,044 (1.42) | 45 (1.54) | 937 (0.44) | 41 (0.02) |  |  |
| **n (outcome)** | 1,892 (0.54) | 3,309 (0.94) | 46 (1.47) | 1,002 (0.29) | 127 (0.04) |  |  |
| **RR (95% CI)** | **1.08 (1.07 to 1.1)** | 1.01 (0.97 to 1.05) | 1.07 (0.97 to 1.19) | 1.00 (0.94 to 1.07) | 0.89 (0.65 to 1.21) | 0.025 (-0.010 to 0.059) | 0.06 (-0.029 to 0.148) |
| **aRR (95% CI)*** | **1.15 (1.11 to 1.19)** | 0.97 (0.93 to 1.01) | 1.08 (0.95 to 1.22) | 0.96 (0.90 to 1.02) | 0.89 (0.65 to 1.22) | 0.018 (-0.019 to 0.056) | 0.074 (-0.023 to 0.171) |
| ***p*_interaction_^b^** | **0.019** | **0.040** | 0.079 | **0.006** | 0.325 | 0.304 | 0.570 |
| Abbreviations: aRR, adjusted risk ratio; CI, confidence interval; ICU, intensive care unit; RR, unadjusted risk ratio.  Bold values indicate statistically significant associations (i.e., 95% CI does not cross 1).  * Risk ratios and length of stay adjusted for the month and year of infection onset, child's age and biological sex, type of health insurance cover, region, population density of residential metropolitan statistical area, mean dew point temperature, and mean temperature.  ^a^ Length of stay in hospital (in days).  ^b^ Referent: Children unvaccinated against influenza. | | | | | | | |

**S5 Table**. Risk ratios and length of stay in hospital for severe acute respiratory infection outcomes associated with an interquartile range (3.34 µg/m^3^) increase in PM_2.5_ exposure among young children aged <5 years, by population density of residential metropolitan statistical area.

|  | **Antiviral prescription claim** | **Hospital admission** | **Hospital readmission** | **ICU admission** | **Mechanical ventilation** | **Length of stay in hospital (hospital-admitted children)^a^** | **Length of stay in hospital (ICU-admitted children)^a^** |
| --- | --- | --- | --- | --- | --- | --- | --- |
| *Population density: Quartile 1 (lowest)* | | | | | | | |
| **N (children)** | 19,059 | 19,059 | 361 | 19,059 | 19,059 | 376 | 70 |
| **N (episodes)** | 31,665 | 31,665 | 387 | 31,665 | 31,665 | 405 | 75 |
| **n (children with outcome)** | 180 (0.94) | 376 (1.97) | 5 (1.39) | 70 (0.37) | 4 (0.02) |  |  |
| **n (outcome)** | 181 (0.57) | 405 (1.28) | 5 (1.29) | 75 (0.24) | 5 (0.02) |  |  |
| **RR (95% CI)** | 0.97 (0.79 to 1.19) | 0.94 (0.86 to 1.03) | 1.30 (0.59 to 2.84) | 1.07 (0.93 to 1.24) | 1.05 (0.82 to 1.35) | 0.060 (-0.139 to 0.259) | 0.065 (-0.135 to 0.264) |
| **aRR (95% CI)*** | 0.99 (0.81 to 1.22) | 0.96 (0.88 to 1.04) | 1.25 (0.31 to 5.08)^c^ | 1.07 (0.93 to 1.24) | 1.03 (0.78 to 1.35) | 0.055 (-0.099 to 0.209) | 0.024 (-0.134 to 0.181) |
| ***p*_interaction_** | [Reference] | [Reference] | [Reference] | [Reference] | [Reference] | [Reference] | [Reference] |
| *Population density: Quartile 2 (second lowest)* | | | | | | | |
| **N (children)** | 40,662 | 40,662 | 638 | 40,662 | 40,662 | 672 | 155 |
| **N (episodes)** | 68,888 | 68,888 | 681 | 68,888 | 68,888 | 724 | 163 |
| **n (children with outcome)** | 387 (0.95) | 672 (1.65) | 13 (2.04) | 155 (0.38) | 7 (0.02) |  |  |
| **n (outcome)** | 387 (0.56) | 724 (1.05) | 13 (1.91) | 163 (0.24) | 19 (0.03) |  |  |
| **RR (95% CI)** | **1.05 (1.03 to 1.08)** | 1.00 (0.95 to 1.06) | 0.69 (0.42 to 1.15) | **1.07 (1.04 to 1.10)** | 0.66 (0.25 to 1.75) | 0.055 (-0.008 to 0.119) | 0.018 (-0.067 to 0.103) |
| **aRR (95% CI)*** | 1.05 (0.98 to 1.12) | 0.98 (0.92 to 1.05) | 0.65 (0.40 to 1.03) | **1.07 (1.03 to 1.10)** | 0.59 (0.21 to 1.69) | 0.059 (-0.006 to 0.124) | 0.045 (-0.059 to 0.149) |
| ***p*_interaction_^b^** | 0.538 | 0.402 | 0.196 | 0.987 | 0.396 | 0.995 | 0.831 |
| *Population density: Quartile 3 (second highest)* | | | | | | | |
| **N (children)** | 111,146 | 111,146 | 1,620 | 111,146 | 111,146 | 1,696 | 477 |
| **N (episodes)** | 190,468 | 190,468 | 1,759 | 190,468 | 190,468 | 1,863 | 519 |
| **n (children with outcome)** | 1,038 (0.93) | 1,696 (1.53) | 26 (1.60) | 477 (0.43) | 17 (0.02) |  |  |
| **n (outcome)** | 1,042 (0.55) | 1,863 (0.98) | 27 (1.53) | 519 (0.27) | 67 (0.04) |  |  |
| **RR (95% CI)** | **1.10 (1.05 to 1.15)** | **1.08 (1.03 to 1.13)** | 1.11 (0.85 to 1.45) | 1.07 (0.97 to 1.17) | 0.72 (0.40 to 1.30) | **0.078 (0.002 to 0.155)** | **0.106 (0.003 to 0.210)** |
| **aRR (95% CI)*** | 1.02 (0.95 to 1.09) | 1.02 (0.97 to 1.08) | 1.17 (0.86 to 1.59) | 1.01 (0.91 to 1.13) | 0.78 (0.42 to 1.44) | **0.087 (0.011 to 0.162)** | 0.109 (-0.0001 to 0.218) |
| ***p*_interaction_^b^** | 0.600 | 0.062 | 0.777 | 0.684 | 0.237 | 0.815 | 0.575 |
| *Population density: Quartile 4 (highest)* | | | | | | | |
| **N (children)** | 202,981 | 202,981 | 2,795 | 202,981 | 202,981 | 2,944 | 994 |
| **N (episodes)** | 346,279 | 346,279 | 2,992 | 346,279 | 346,279 | 3,202 | 1,070 |
| **n (children with outcome)** | 2,090 (1.03) | 2,944 (1.45) | 37 (1.32) | 994 (0.49) | 35 (0.02) |  |  |
| **n (outcome)** | 2,105 (0.61) | 3,202 (0.92) | 38 (1.27) | 1,070 (0.31) | 93 (0.03) |  |  |
| **RR (95% CI)** | **1.09 (1.08 to 1.10)** | **1.04 (1.02 to 1.07)** | 0.95 (0.71 to 1.29) | 1.04 (0.99 to 1.09) | **1.08 (1.02 to 1.16)** | 0.016 (-0.026 to 0.059) | 0.019 (-0.062 to 0.100) |
| **aRR (95% CI)*** | **1.15 (1.12 to 1.18)** | 1.01 (0.97 to 1.04) | 0.89 (0.61 to 1.29) | 1.00 (0.94 to 1.07) | **1.09 (1.01 to 1.18)** | 0.015 (-0.030 to 0.061) | 0.026 (-0.061 to 0.114) |
| ***p*_interaction_^b^** | 0.294 | 0.216 | 0.466 | 0.399 | 0.713 | 0.730 | 0.853 |
| Abbreviations: aRR, adjusted risk ratio; CI, confidence interval; ICU, intensive care unit; RR, unadjusted risk ratio.  Bold values indicate statistically significant associations (i.e., 95% CI does not cross 1).  * Risk ratios and length of stay adjusted for the month and year of infection onset, child's age and biological sex, influenza vaccination status, type of health insurance cover, region, mean dew point temperature, and mean temperature.  ^a^ Length of stay in hospital (in days).  ^b^ Referent: Population density: Quartile 1 (lowest).  ^c^ Could not adjust for child’s biological sex. | | | | | | | |

**S6 Table**. Risk ratios and length of stay in hospital for severe acute respiratory infection outcomes associated with an interquartile range (3.34 µg/m^3^) increase in PM_2.5_ exposure among young children aged <5 years: sensitivity analyses.

|  | **Antiviral prescription claim** | **Hospital admission** | **Hospital readmission** | **ICU admission** | **Mechanical ventilation** | **Length of stay in hospital (hospital-admitted children)^a^** | **Length of stay in hospital (ICU-admitted children)^a^** |
| --- | --- | --- | --- | --- | --- | --- | --- |
| *Children with one year of continuous health insurance coverage prior to date of infection* | | | | | | | |
| **N (children)** | 177,330 | 177,330 | 2,059 | 177,330 | 177,330 | 2,158 | 664 |
| **N (episodes)** | 300,737 | 300,737 | 2,230 | 300,737 | 300,737 | 2,371 | 715 |
| **n (children with outcome)** | 585 (0.33) | 2,158 (1.22) | 31 (1.51) | 664 (0.37) | 40 (0.02) |  |  |
| **n (outcome)** | 590 (0.20) | 2,371 (0.79) | 33 (1.48) | 715 (0.24) | 127 (0.04) |  |  |
| **RR (95% CI)** | 1.00 (0.94 to 1.07) | 0.96 (0.91 to 1.01) | 1.04 (0.74 to 1.46) | 1.01 (0.93 to 1.09) | 0.84 (0.59 to 1.19) | **0.119 (0.044 to 0.194)** | **0.150 (0.059 to 0.241)** |
| **aRR (95% CI)*** | 0.92 (0.82 to 1.02) | **0.94 (0.89 to 0.98)** | 1.06 (0.75 to 1.51) | 0.98 (0.91 to 1.06) | 0.86 (0.63 to 1.18) | **0.125 (0.046 to 0.204)** | **0.151 (0.065 to 0.238)** |
| *Children with an onset of infection any time during the year* | | | | | | | |
| **N (children)** | 398,880 | 398,880 | 6,005 | 398,880 | 398,880 | 6,293 | 1,879 |
| **N (episodes)** | 760,780 | 760,780 | 6,570 | 760,780 | 760,780 | 7,001 | 2,066 |
| **n (children with outcome)** | 3,705 (0.93) | 6,293 (1.58) | 89 (1.48) | 1,879 (0.47) | 71 (0.02) |  |  |
| **n (outcome)** | 3,725 (0.49) | 7,001 (0.92) | 92 (1.40) | 2,066 (0.27) | 259 (0.03) |  |  |
| **RR (95% CI)** | **1.06 (1.05 to 1.08)** | 1.01 (0.99 to 1.04) | 0.97 (0.80 to 1.17) | **1.04 (1.01 to 1.08)** | 1.02 (0.88 to 1.18) | **0.035 (0.004 to 0.066)** | 0.035 (-0.018 to 0.088) |
| **aRR (95% CI)*** | **1.09 (1.05 to 1.14)** | 0.99 (0.97 to 1.02) | 0.98 (0.81 to 1.18) | 1.02 (0.98 to 1.06) | 1.02 (0.88 to 1.18) | **0.036 (0.003 to 0.068)** | 0.042 (-0.013 to 0.098) |
| Abbreviations: aRR, adjusted risk ratio; CI, confidence interval; ICU, intensive care unit; RR, unadjusted risk ratio.  Bold values indicate statistically significant associations (i.e., 95% CI does not cross 1).  * Risk ratios and length of stay adjusted for the month and year of infection onset, child's age and biological sex, influenza vaccination status, type of health insurance cover, region, population density of residential metropolitan statistical area, mean dew point temperature, and mean temperature.  ^a^ Length of stay in hospital (in days). | | | | | | | |

**S7 Table**. Risk ratios and length of stay in hospital for severe acute respiratory infection outcomes associated with an interquartile range (3.34 µg/m^3^) increase in PM_2.5_ exposure among young children aged <5 years, by definition of infection.

|  | **Antiviral prescription claim** | **Hospital admission** | **Hospital readmission** | **ICU admission** | **Mechanical ventilation** | **Length of stay in hospital (hospital-admitted children)^a^** | **Length of stay in hospital (ICU-admitted children)^a^** |
| --- | --- | --- | --- | --- | --- | --- | --- |
| *Acute respiratory infection (principal and additional diagnosis fields)* | | | | | | | |
| **N (children)** | 440,132 | 440,132 | 6,558 | 440,132 | 440,132 | 6,994 | 2,097 |
| **N (episodes)** | 846,775 | 846,775 | 7,166 | 846,775 | 846,775 | 7,803 | 2,295 |
| **n (children with outcome)** | 4,382 (1.00) | 6,994 (1.59) | 100 (1.52) | 2,097 (0.48) | 90 (0.02) |  |  |
| **n (outcome)** | 4,411 (0.52) | 7,803 (0.92) | 102 (1.42) | 2,295 (0.27) | 264 (0.03) |  |  |
| **RR (95% CI)** | **1.08 (1.07 to 1.09)** | 1.02 (0.999 to 1.04) | 0.98 (0.82 to 1.16) | 1.03 (0.99 to 1.07) | 0.98 (0.81 to 1.19) | 0.006 (-0.027 to 0.04) | 0.014 (-0.049 to 0.076) |
| **aRR (95% CI)*** | **1.12 (1.09 to 1.16)** | 0.99 (0.97 to 1.02) | 0.98 (0.81 to 1.18) | 1.00 (0.95 to 1.04) | 0.96 (0.81 to 1.15) | 0.014 (-0.022 to 0.05) | 0.013 (-0.058 to 0.083) |
| *Influenza infection (principal diagnosis field only)* | | | | | | | |
| **N (children)** | 69,632 | 69,632 | 535 | 69,632 | 69,632 | 561 | 121 |
| **N (episodes)** | 74,497 | 74,497 | 538 | 74,497 | 74,497 | 565 | 122 |
| **n (children with outcome)** | 2,988 (4.29) | 561 (0.81) | 6 (1.12) | 121 (0.17) | 3 (0) |  |  |
| **n (outcome)** | 3,003 (4.03) | 565 (0.76) | 6 (1.12) | 122 (0.16) | 3 (0) |  |  |
| **RR (95% CI)** | **1.08 (1.05 to 1.11)** | 1.01 (0.91 to 1.12) | 1.33 (0.69 to 2.59) | 0.98 (0.73 to 1.30) | 0.79 (0.58 to 1.08) | **0.179 (0.009 to 0.350)** | **0.220 (0.012 to 0.427)** |
| **aRR (95% CI)*** | **1.10 (1.05 to 1.15)** | 0.98 (0.90 to 1.07) | 1.39 (0.37 to 5.24)^b^ | 0.91 (0.69 to 1.20) | 0.88 (0.57 to 1.35)^c^ | **0.208 (0.015 to 0.400)** | **0.331 (0.114 to 0.548)** |
| *Influenza infection (principal and/or additional diagnosis fields)* | | | | | | | |
| **N (children)** | 88,518 | 88,518 | 718 | 88,518 | 88,518 | 756 | 181 |
| **N (episodes)** | 96,484 | 96,484 | 726 | 96,484 | 96,484 | 765 | 182 |
| **n (children with outcome)** | 3,757 (4.24) | 756 (0.85) | 9 (1.25) | 181 (0.20) | 5 (0.01) |  |  |
| **n (outcome)** | 3,779 (3.92) | 765 (0.79) | 9 (1.24) | 182 (0.19) | 5 (0.01) |  |  |
| **RR (95% CI)** | **1.08 (1.06 to 1.10)** | 0.99 (0.90 to 1.08) | 0.88 (0.28 to 2.77) | 0.92 (0.73 to 1.16) | **0.45 (0.21 to 0.97)** | 0.817 (0.474 to 1.159) | 0.193 (-0.005 to 0.392) |
| **aRR (95% CI)*** | **1.11 (1.07 to 1.15)** | 0.96 (0.88 to 1.03) | 0.78 (0.19 to 3.19)^b^ | 0.86 (0.69 to 1.07) | 0.40 (0.14 to 1.18)^d^ | 0.141 (-0.017 to 0.299) | **0.290 (0.068 to 0.512)** |
| Abbreviations: aRR, adjusted risk ratio; CI, confidence interval; ICU, intensive care unit; RR, unadjusted risk ratio.  Bold values indicate statistically significant associations (i.e., 95% CI does not cross 1).  * Risk ratios and length of stay adjusted for the month and year of infection onset, child's age and biological sex, influenza vaccination status, type of health insurance cover, region, population density of residential metropolitan statistical area, mean dew point temperature, and mean temperature.  ^a^ Length of stay in hospital (in days).  ^b^ Could not adjust for type of health insurance coverage and region.  ^c^ Could not adjust for child’s biological sex, influenza vaccination status, type of health insurance coverage, and region.  ^d^ Could not adjust for child’s biological sex, type of health insurance coverage, and region. | | | | | | | |
